# Role of Mesh Pore Size in Dynamic Membrane Bioreactors

**DOI:** 10.3390/ijerph18041472

**Published:** 2021-02-04

**Authors:** Pompilio Vergine, Carlo Salerno, Barbara Casale, Giovanni Berardi, Alfieri Pollice

**Affiliations:** Water Research Institute of the National Research Council of Italy (IRSA CNR), Viale F. De Blasio, 5-70132 Bari, Italy; pompilio.vergine@ba.irsa.cnr.it (P.V.); barbara.casale@ba.irsa.cnr.it (B.C.); giovanni.berardi@ba.irsa.cnr.it (G.B.); alfieri.pollice@ba.irsa.cnr.it (A.P.)

**Keywords:** biological process, Membrane BioReactor, mesh filtration, pore size, wastewater treatment

## Abstract

Two identical bench-scale Self-Forming Dynamic Membrane BioReactors (SFD MBR) were set-up and operated for the treatment of real urban wastewater. The two bioreactors were equipped with meshes of different mesh pore size. Meshes having pore size values of 20 and 50 µm were tested under solid retention time (SRT) of 15 d, whereas meshes with 50 and 100 µm pore sizes were compared under SRT of 50 d. The results of long-term experiments showed very good overall performances by all systems at the steady state. High flux (in the range 61–71 L m^−2^ h^−1^) and very good effluent quality were obtained, with average suspended solids and chemical oxygen demanding values below 10 mg L^−1^ and 35 mg L^−1^, respectively. The mesh pore size did not have a major influence on the average cleaning frequency. However, the pore size affected the effluent quality in correspondence of two particular conditions: (i) immediately after mesh cleaning; and (ii) during operation under high suction pressures (mesh clogging not promptly removed through cleaning). Moreover, the mesh cleaning frequency was observed to be dependent on the SRT. In tests with 50 d SRT, the cleaning requirements were very low (one every five days), and this limited the influence of the mesh pore size on the effluent quality. In conclusion, in SFD MBR, the role of the mesh pore size on the effluent quality may be more or less relevant depending on the operating conditions that directly influence the Dynamic Membrane formation.

## 1. Introduction

The Membrane BioReactor (MBR) is a wastewater treatment technology resulting from the integration of membrane filtration into the activated sludge process, and it is one of the most important innovations developed in this field [1]. The main benefit of the MBR is the production of high-quality effluents without any further treatment. However, 30 years of application of ultrafiltration-based MBR have shown that membrane fouling may rapidly reduce the system’s productivity, i.e., the flow rate produced per unit of pressure applied, and increase its management costs [2]. This, together with the relatively high costs and fragility of polymeric membranes, limits the application of the MBR in large-scale urban wastewater treatment plants (WWTPs).

An innovative approach that aims to avoid these drawbacks is represented by the Self-Forming Dynamic Membrane BioReactor (SFD MBR), based on the replacement of polymeric ultrafiltration membranes with relatively coarse filtering supports (pore size usually in the range 10–100 µm) made of low-cost materials. During the filtration process, the accumulation of mixed liquor-activated sludge on the backing support due to external forces (i.e., suction, gravity filtration) results in the growth of a biological cake layer, which is the Dynamic Membrane (DM) [3]. The pores within the DM are much smaller compared to those of the filtering support, and therefore the filtration through the DM can lead to a treated wastewater of good quality, with values of turbidity close to 1 nephelometric turbidity unit (NTU) [4,5,6]. The system’s productivity can be stably maintained through simple mechanical cleaning procedures [6]. The low-cost materials, the low energy needs, and the simple care make the SFD MBR a good substitute for conventional MBR.

Results of several investigations indicate that the SFD MBR technology still needs to be optimized in terms of operating conditions [7]. Several studies have emphasized the physical characteristics of SFD MBR, but their results are sometimes contradictory [3,8,9,10,11,12,13,14]. Salerno and co-authors [10] compared 20 and 50 µm pore size values under different mesh scouring intensities, and found that optimal hydrodynamic conditions strongly limit the influence of the pore size on the effluent quality. Saleem and co-authors [11] compared pore size values in the range 10–200 µm and, whereas no significant differences were observed during short filtration tests with anaerobic sludge, when the same set-up was applied in a long-term bioreactor operation under anoxic-aerobic conditions, the pore size was a critical factor to achieve satisfactory filtration performances [12]. Cai and co-authors [13] tested five pore sizes values (1, 5, 10, 25, and 50 µm) and found that the relevance of the pore size on both the productivity and the effluent turbidity depended on the other operating conditions, as indicated by the partially different results observed during short filtration tests and long-term operation. Ersahin and co-authors [14] compared 10 and 40 µm using materials characterized by different yarn types (mono-monofilament, mono-multifilament and staple) and only in one case out of three did the pore size affect the productivity. These studies indicate that the effect of the filtering support pore size on the system performances depends on other operating parameters, and the interconnections among all the influencing factors are not yet understood. Therefore, bridging this knowledge gap would be important on the way to the improvement of the SFD MBR.

The aim of this study was to evaluate the influence of the mesh pore size on DM performance under different solid retention time (SRT) values. For this purpose, the study compares the results obtained in four long-term tests performed with bench-scale systems equipped with nylon meshes for the treatment of real urban wastewater. Meshes having pore size values of 20 and 50 µm were tested under SRT of 15 d, and meshes with 50 and 100 µm pore sizes were compared under SRT of 50 d, all under the same operating conditions, including temperature, air scouring, hydraulic retention time (HRT), volumetric loading rate (VLR) and flux.

## 2. Materials and Methods

### 2.1. Bioreactor Operation

**Figure 1:**
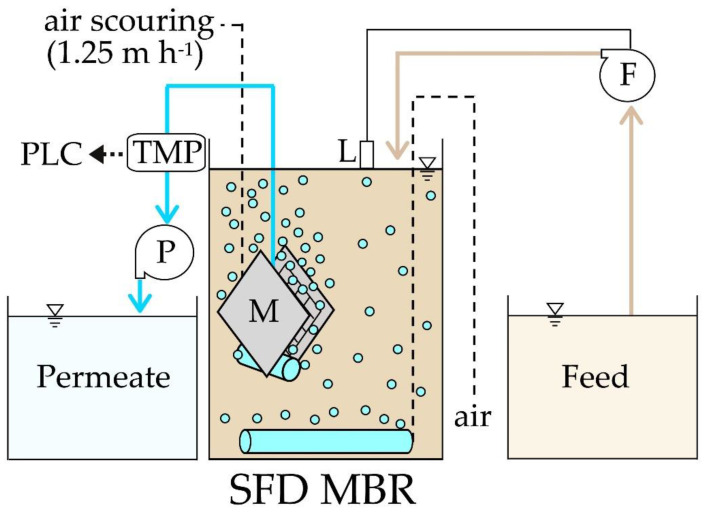
Plant scheme of the Self-Forming Dynamic Membrane BioReactor (SFD MBR). P, permeate pump; TMP, manometer with transmembrane pressure transducer connected to a PLC; M, filtration module; F, feed pump; L, level control connected to F.

Two 4 L SFD MBR, identical except for the filtering support pore size, were operated in parallel under the same experimental conditions for the treatment of real urban wastewater, whose characteristics are described in Table 1. In each reactor, a submerged filtration module made of nylon mesh (mono-monofilament woven fabric, Nitex^®^, Sefar AG, Heiden, Switzerland) was used to support the growth of the DM. Each filtration module was composed of two parallel filtering surfaces of 6 × 6 cm, resulting in a total filtering surface of 72 cm^2^. A fine bubble diffuser was placed at the bottom of the module ensuring a continued air scouring of both the filtering surfaces with a flow rate of 9.0 L_air_ h^−1^ (equal to 1.25 m^3^ h^−1^ per m^2^ of surface). The dissolved oxygen (DO) concentration was kept in the range 2–4 mgO_2_ L^−1^ through an air diffuser placed at the bottom of the reactor, which also ensured the sludge mixing. A peristaltic pump (IPCR6, Espango-Teknofluor s.r.l., Milan, Italy) extracted the permeate from the filtration module at the set flux. The feed pump was activated by a level control to maintain a constant volume in the biological tank. The temperature of the biological tank was maintained at 20 °C. An optical glycerine manometer directly linked to a pressure transducer (VAL.CO s.r.l., S. Ilario di Nerviano, Italy) was used to measure the transmembrane pressure (TMP) and to record it every 15 min using a programmable logic controller (PLC) (Intesis s.r.l., Bari, Italy). Figure 1 shows a schematization of the experimental plant. When TMP was found beyond the value of −100 mbar, the module was removed from the reactor and the mesh surfaces were cleaned by jet rinsing with tap water for approximately 5 min. Overall, the cleaning procedure required the interruption of the bioreactor operation for about 15 min. Some pictures of the nylon flat sheet support, mature DM. and samples of feed and permeate are available as Supplementary Materials.

In total, 4 runs were carried out, each one named as Rx_y, with x and y representing the adopted mesh pore size (µm) and SRT (d), respectively. The steady state operating conditions of the 4 runs are described in Table 2. Some results from two of these runs were discussed in a previous paper focused on the role of the SRT on process efficiency [15]. In the present investigation, they were further elaborated and compared with new experimental data for evaluating the role of mesh pore size.

### 2.2. Monitoring and Analyses

The main water quality parameters, including turbidity, total suspended solids (TSS), chemical oxygen demand (COD), total nitrogen (TN), ammonium, nitrite, nitrate, and total phosphorus (TP) were measured both in the feed (once per week) and in the effluent (24 h average samples, three per week). The permeate turbidity was measured five times per week. The concentration of suspended biomass, i.e., the mixed liquor suspended solids (MLSS), and the concentration of dissolved oxygen (DO) in the mixed liquor, were measured three times per week.

All physicochemical parameters were analyzed according to the Standard Methods for the Examination of Water and Wastewater [16]. Turbidity was measured with a 2100P turbidimeter (HACH, Loveland, CO, USA). A luminescent DO probe (HACH, Loveland, CO, USA) was used for the measurement of DO concentration. Electrical conductivity, temperature and pH were monitored through a InoLab^®^ Multi 9420 IDS (WTW, Weilheim, Germany).

The effluent turbidity values observed at the steady state were statistically analyzed using the Mann–Whitney U Test. Four datasets were considered for each run. The “all data” dataset comprised all values measured during the run, which were also divided in three groups, the “1st day”, “2nd day”, and “>2 days” datasets determined by the time between mesh cleaning and effluent sampling.

## 3. Results

### 3.1. Average Performance

Table 3 shows the average values of the main parameters of the SFD MBR at the steady state. Moreover, in all the experimental runs, ammonium and nitrite levels in the effluent were always below 1 mgN L^−1^, indicating complete nitrification. Considering all runs, the bench scale SFD MBR effectively removed suspended solids and organic matter, producing a very good quality effluent. The average effluent quality observed during the four runs confirms previous studies on aerobic SFD MBR treating municipal wastewater [5,17,18,19,20,21]. In this study, it was remarkable that high flux (Table 2) and low effluent turbidity (Table 3) were stably obtained during long-term experiments. This aspect contributes to demonstrating the reliability of the proposed technology.

### 3.2. 15 D SRT

**Figure 2:**
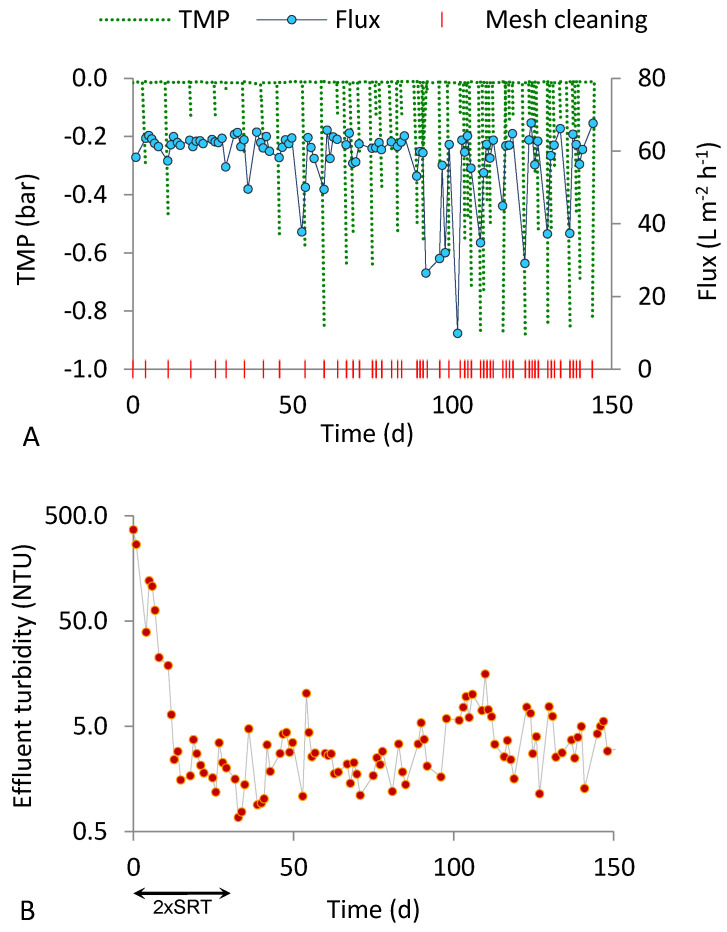
Results obtained in run R50_15 (50 µm pore size, 15 d SRT). (**A**) Flux, TMP and mesh cleaning; (**B**) effluent turbidity.

**Figure 3:**
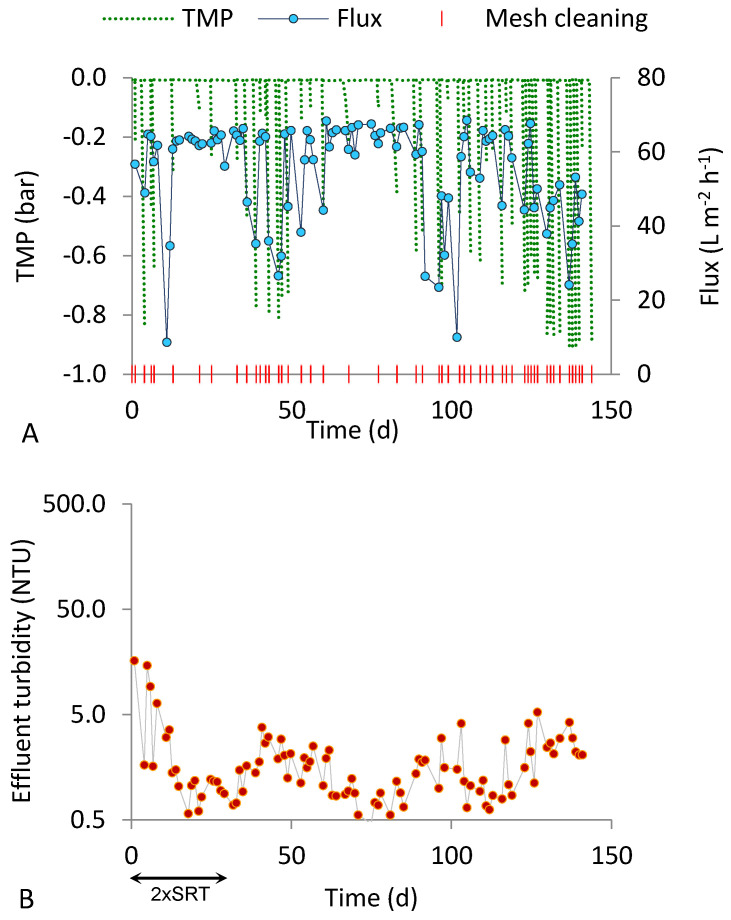
Results obtained in run R20_15 (20 µm pore size, 15 d SRT). (**A**) Flux, TMP and mesh cleaning; (**B**) effluent turbidity.

Figure 2 and Figure 3 show the fluxes produced and the effluent turbidity values observed during the two tests performed at an SRT of 15 d (R20_15 and R50_15). The cleaning requirements were variable at the steady state, from one to five times per week. In the periods with the highest cleaning requirements, TMP increased during unobserved periods, triggering a decrease in the permeate flux. Figure 2A and Figure 3A show the accordance between low peaks of flux and TMP values, except for the reduction in the flux caused by temporary interruptions due to failed or degraded plant components (Figure 2A, at around day 100; Figure 3A, at around day 10 and day 100).

The charts of effluent turbidity for both runs (Figure 2 and Figure 3) reveal that the permeate quality improved after about one week from the beginning. However, the solid retention performance of the two runs conducted at SRT of 15 d were different. The average permeate turbidity obtained with the 50 µm mesh at the steady state was more than twice higher than that obtained with the 20 µm mesh (Table 3). The smaller pore size may have contributed to maintain very good filtration performance along the entire steady state period (maximum effluent turbidity of 5.3 NTU). Additionally, during the transient period (first 30 d after start-up), the effluent turbidity was higher, on average, in run R50_15. However, the relatively high variability of the effluent turbidity data, as indicated by the standard deviations in Table 3, does not enable the drawing of conclusions about the influence of the mesh pore size under 15 d SRT; the datasets will be statistically compared in Section 3.3.

### 3.3. 50 D SRT

**Figure 4:**
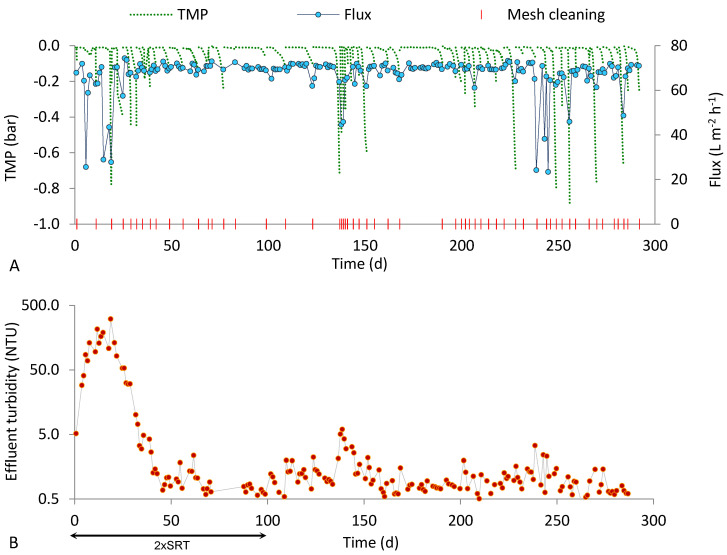
Results obtained in run R50_50 (50 µm pore size, 50 d SRT). (**A**) Flux, TMP and mesh cleaning; (**B**) effluent turbidity.

**Figure 5:**
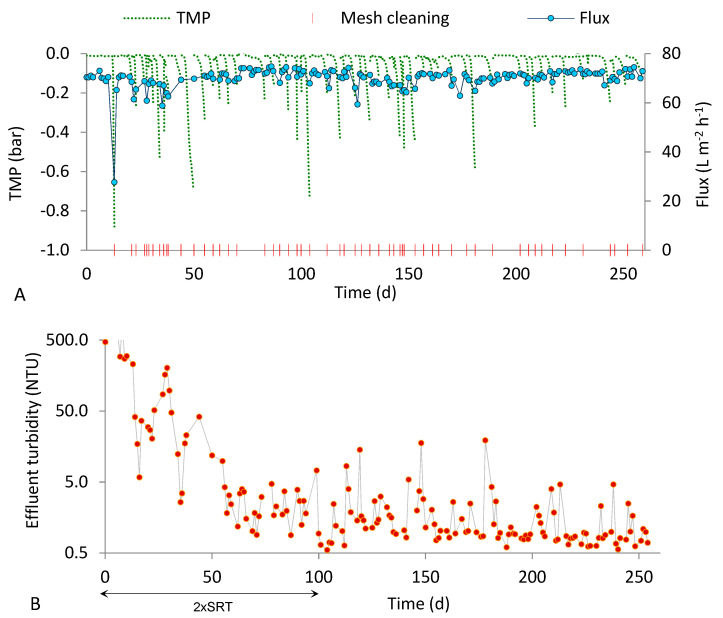
Results obtained in run R100_50 (100 µm pore size, 50 d SRT). (**A**) Flux, TMP and mesh cleaning; (**B**) effluent turbidity.

The SFD MBR performance during the two runs carried out at 50 d SRT were similar (Figure 4 and Figure 5). In both runs R50_50 and R100_50, the filtering supports had low cleaning requirements (fewer than two cleanings per week), which allowed for a stable productivity. This occurred despite the higher MLSS concentration obtained at an SRT of 50 d with respect to SRT of 15 d, confirming that the system’s productivity (strictly related to the cleaning frequency) depended more on the properties of the suspended biomass than on its concentration. Previous studies performing short filtration tests indicated that the DM productivity decreased at higher MLSS concentration [11,22,23], whereas when this parameter was varied as a result of changes in biological parameters (SRT or VLR) that affect sludge characteristics; the opposite effect was observed [3,18,24]. In particular, the content of extracellular polymeric substances EPS was shown to be reduced by increasing the SRT [25,26].

The effluent turbidity had approximately the same trend in both runs. Due to the high SRT of 50 d, the activated sludge needed a long adaptation period before forming an effective biological filter on the mesh, but then the DM showed very good performance during the entire steady state period, with the 90th percentile of the effluent turbidity below 3 NTU in both runs. However, effluent turbidity values above 10 NTU were occasionally observed during run R100_50. This is the only noticeable difference between the performance of runs R50_50 and R100_50, and, considering the identical operating conditions of the two tests (which only differed in pore size), it was reasonably due to the different mesh pore sizes (50 vs. 100 µm). Additionally, in this case, as observed at 15 d SRT, a statistical inference was required to compare the effluent turbidity datasets.

### 3.4. Statistical Inference

**Figure 6:**
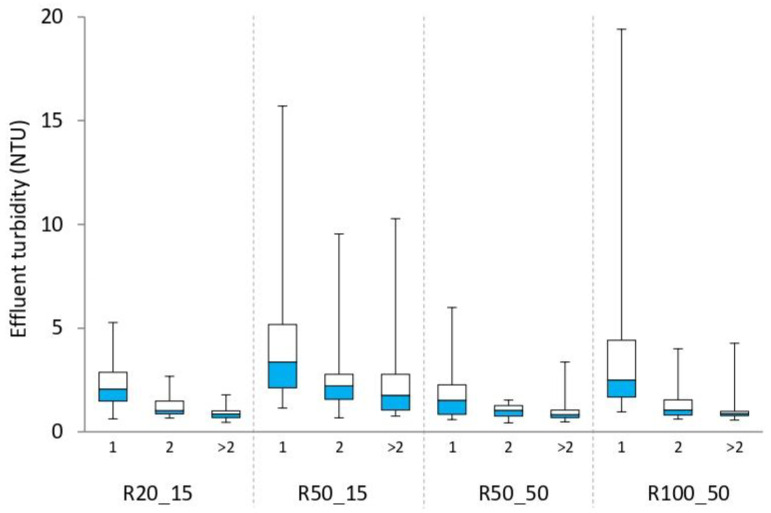
Box plots of the permeate turbidity datasets measured at the steady state, excluding the first transient period equal to twice the SRT. For each test, three classes were distinguished according to the number of days since the last mesh cleaning. Run Rx_y corresponds to “x” mesh pore size (μm) and “y” SRT (d).

For each experiment, the steady state permeated turbidity data (thereby excluding the data related to the first transient period equal to twice the SRT) were divided into three classes according to the number of days between a mesh cleaning and the following sampling. These datasets are shown in Figure 6 as box plots. The results of the statistical comparisons (*p*-values of the Mann–Whitney U test) are listed in Table 4.

The comparison between the “all data” series of runs R20_15 and R50_15 showed that at SRT of 15 d, the pore size had a significant effect on effluent turbidity (Table 4, line 1), with the median value from run R50_15 almost twice the one calculated for run R20_15 (Figure 6). Observing the comparisons between the classes of these two runs, the role of mesh pore size can be better understood. Significant differences were found for the “1st day” series, but not for the “2nd day” data (Table 4, line 1). This suggests that the pore size had a relevant role in the period immediately subsequent to mesh cleaning, but its influence was not so significant once the DM was formed. However, the “>2 days” series from run R20_15 and R50_15 had highly significant differences (Table 4, line 1), with the first run having more stable and lower values than the second run (Figure 6). This is probably related to the rapid TMP increase observed under 15 d SRT, meaning that most of the data from the “>2 days” group of runs run R20_15 and R50_15 corresponded to operation under high suction pressures (data not shown). The latter may have favored the formation of preferential paths in the DM formed on the 50 µm mesh, as previously described [10,13,27], whereas no relevant DM destabilization was observed in the filtering layer of the 20 µm mesh.

Comparing the “all data” series of runs performed with 50 d SRT (R50_50 and R100_50), the effect of the different pore size was observed to be statistically significant (*p*-value < 0.05) but very small, with very similar median turbidity values (Figure 6). Moreover, the comparisons of the classes revealed that the influence of pore size on effluent turbidity was limited to the DM formation stage (i.e., “1st day”), whereas no significant difference was observed with respect to the “2nd day” and “>2 days” classes of runs R50_50 and R100_50 (Table 4, line 2). This means that at 50 d SRT, the solid retention performed by the DM during its maturity stage was not influenced by the pore size of the underlying support.

Furthermore, runs operated under different SRTs and different pore size were also compared. Comparing runs R50_15 and R100_50, the latter had better overall performance (Table 4, line 3; Figure 6), suggesting a predominance of the positive influence of the higher SRT (50 vs. 15 d) over the negative influence of the larger mesh pore size (100 vs. 50 µm). In particular, according to the *p*-values related to the comparisons between the classes (Table 4, line 3), this predominance increased with the time passed from the previous mesh cleaning. This suggests that mesh pore size had a relevant role during the early stage of the DM formation, whereas once the maturity stage was reached, the features of the DM were mostly determined by the activated sludge characteristics (i.e., by the SRT).

Finally, comparing both runs operated at the highest SRT (R50_50 and R100_50) with the run operated with the smallest mesh pore size (R20_15), the former had significantly better overall filtration performance, as shown by the differences among the “all data” series (Table 4, lines 4 and 5; Figure 6). However, comparing the corresponding classes, the median values were very close to each other (Figure 6), and the differences were not statistically significant (Table 4, lines 4 and 5). In previous research, it was observed that the turbidity values during the first day after the filtering support cleaning were higher with respect to the following days [6,8,10,13]. For this reason, the better performance of runs R50_50 and R100_50 with respect to run R20_15 was probably due to the lower cleaning occurrence at higher SRT, which meant a lower number of new DM formation stages. As a matter of fact, in the two tests performed at SRT of 15 d, the mesh cleaning was performed almost every two days (Table 3); therefore, the “1st day” class contained about half of the data, whereas in the three runs operated at higher SRTs, the majority of the data were in the “>2 days” class. We may conclude that the opposite effects of the changes in pore size (from 20 to 50/100 µm) and SRT (from 15 to 50 d) on the performance of the self-formed DM practically offset each other, but at higher SRT the SFD MBR operated with the DM in its maturity stage for longer, thereby enhancing the overall solid retention performance.

Overall, the statistical analysis indicated the role of the mesh pore size under the different phases of the DM development (formation, stability and, eventually, mesh fouling), and also showed that the pore size can be a key or a negligible factor, depending on the biological operating parameters that affect sludge characteristics, such as the SRT.

## 4. Discussion

This study focused on the evolution of permeate flux and effluent turbidity over time as indicators for the evaluation of integrated process performance under different biological process conditions (SRT) and filtration features (pore size). Results of the main parameters characterizing the integration of biological and filtration processes are also presented together with other effluent quality parameters (TSS, COD, ammonium, and nitrite) and process operation characteristics (MLSS, cleaning frequency) (Table 3).

The two runs with the higher SRT of 50 d were characterized by higher MLSS concentrations, lower cleaning requirements, and lower effluent turbidity values with respect to those with 15 d SRT (Table 3). The higher SRT resulted in better overall filtration performances, as previously discussed [15].

The mesh pore size was observed to have a limited influence on the average cleaning frequency, as can be noticed by comparing the couples of runs performed at the same SRT, i.e., R20_15 vs. R50_15 and R50_50 vs. R100_50 (Table 3). Higher cleaning requirements at lower mesh pore sizes were observed by the authors in a previous study conducted under similar experimental conditions, except for the operating flux [10]. These different results may be due to the higher flux adopted (90 L m^−2^ h^−1^) and the shorter test period (approximately 60 d per each run). As a matter of fact, the findings of the present study suggest that long-term experiments are necessary to test the influence of operating parameters on the performance of the SFD MBR. During each experimental run, the cleaning frequency was very variable, also under steady operating conditions. This suggests that fluctuations in the physical and biochemical characteristics of the self-forming DM may occur as a consequence of unpredictable factors, such as small changes in the composition of the urban wastewater, towards which these biological layers seem to be especially sensitive [28].

Regarding the effluent quality, the mesh pore size had a relevant role only during two particular phases of the operation: (i) the first period after mesh cleaning (i.e., during DM formation); and (ii) under high suction pressures due to excessive DM accumulation not promptly removed through cleaning (i.e., pressure increase during nights or weekends, possibly causing cracks and preferential paths through the DM). The latter happened more frequently during runs with 15 d SRT, whereas at higher SRT the system worked mostly with a DM in its maturity stage and with a TMP close to zero, showing a lower mesh clogging tendency. Under these conditions, the solid retention performance was largely independent of the mesh pore size. We can conclude that the effluent quality under the tested conditions primarily depends on the steady state-activated sludge features (which in turn are affected by the overall operating conditions, including the SRT). The influence of the mesh pore size was relevant only under specific conditions, corresponding to transient phenomena for the biomass forming the DM, i.e., during DM formation stage, under conditions that generate the DM destabilization, such as high suction pressures, or under unstable biological process, which can cause EPS production.

Table 5 provides an overview of the state of the art on the role of the filtering support pore size in DM bioreactors. Overall, under both aerobic and anaerobic conditions, higher pore size values result in higher productivity and lower effluent quality. However, the findings summarized in Table 5 concur to indicate that the influence of this parameter on the system performances is strongly affected by other factors; from the literature studies, it is not possible to draw a general conclusion on the role of the pore size in DM bioreactors. Saleem [11] and Cai [13] with respective colleagues obtained different results depending on the type of tests performed (short filtration tests vs. long term bioreactor operation) and oxidative conditions, which are two aspects that clearly affect sludge characteristics. Ersahin et al. [14] highlighted the importance of the material structure, suggesting that the interaction between sludge and filtering support cannot be simplified in a two-dimensions model. All the findings reported in Table 5 suggest that sludge characteristics, flux, TMP, and hydrodynamic conditions may affect the DM formation process to a greater extent with respect to the filtering support characteristics. Future research should study the role of the pore size in conjunction with other influencing factors, in order to explore the possible combined effects. Within this context, the findings of this work show two main results: (i) the SRT can influence the filtration performance to a greater extent than the filtering support pore size; and (ii) the latter can be a relevant factor when the system works under unstable conditions for the DM. Indeed, under the operating conditions tested (VLR of 0.9–1.1 gCOD L^−1^ d^−1^, flux of 61–71 L m^−2^ h^−1^, pore size of 20–100 µm, and SRT of 15 and 50 d), the filtering support pore size was not the most important factor affecting the performance of the system, even though a tighter mesh promoted the DM formation and enhanced its stability under high suction pressures. In order to obtain good and stable SFD MBR performances, attention must be paid to the biological operating conditions and to their interaction with the physical parameters; therefore, the choice of the filtering support pore size should be adapted to the specific biomass characteristics.

## 5. Conclusions

During a long-term study conducted with two pairs of bench-scale SFD MBR, four runs were performed in order to evaluate the role of the mesh pore size (in the range 20–100 µm) under different SRTs (15 and 50 d). Overall, high flux (in the range 61–71 L m^−2^ h^−1^) and very good effluent quality were obtained, with average TSS and COD values below 10 mg L^−1^ and 35 mg L^−1^, respectively. The results obtained at the steady state evidenced that the mesh pore size had negligible effects on the cleaning requirements and a small influence on the effluent quality. The latter was limited to the periods of operation with DM under formation stage, or under high suction pressures. On the contrary, when the DM was in its maturity stage and the TMP was close to zero, the effluent quality was observed to be independent of the mesh pore size.

The outcomes of this study show that a rapid formation and a good stability of the DM are generally obtained as a result of steady biological and physical operating conditions. In this regard, high SRT values play a primary role. On the other hand, when this stability cannot be ensured (e.g., under short SRT and/or variable feed conditions), a relatively small pore size of the supporting medium (20 µm or close to it) can favor the DM development.

For full scale applications, the high variability of the operating conditions can play an important role in the system performance. In particular, feeding, temperature and DO concentration relevantly affect the sludge characteristics and, consequently, all the findings related to this technology need to be tested and validated under variable operating conditions. On the other side, the automation of the cleaning systems can be easier in large-scale plants, thereby limiting the possible reduction in productivity and the consequent system instability correlated to the operation with a partially clogged filtering support.

## Figures and Tables

**Table 1:** Values of the main physicochemical parameters of the real urban wastewater treated by the SFD MBR.

Parameter	10th Percentile	Median	90th Percentile
pH	7.0	7.5	7.9
Electrical conductivity (µS cm^−1^)	1831	2340	2656
Chemical oxygen demand (mgO_2_ L^−1^)	357	389	410
Total suspended solids (mg L^−1^)	121	186	279
Total nitrogen (mg L^−1^)	46.3	61.5	71.3
N–NH_4_^+^ (mg L^−1^)	32.4	43.0	52.1
Total phosphorus (mg L^−1^)	9.1	12.8	17.2

**Table 2:** Adopted mesh pore sizes and operating conditions in all tested runs.

Run	Mesh Pore Size (µm)	SRT (d)	Flow Rate (*) (L d^−1^)	Flux (*) (L m^−2^ h^−1^)	HRT (*) (h)	VLR (*) (gCOD L^−1^ d^−1^)	Test Time (d)
R20_15	20	15	10.6	61.3	9.5	0.93	141
R50_15	50	15	10.6	61.3	9.5	0.93	141
R50_50	50	50	11.7	67.7	8.6	1.09	293
R100_50	100	50	12.3	71.2	8.2	1.14	253

(*) Median values at steady state, i.e., excluding the transient period equal to twice the SRT.

**Table 3:** Average values of the main parameters at the steady state for each run (i.e., excluding the transient period equal to twice the SRT). Run Rx_y corresponds to “x” mesh pore size (μm) and “y” SRT (d).

Parameter	R20_15	R50_15	R50_50	R100_50
MLSS (g L^−1^)	2.3 ± 0.6	2.0 ± 0.7	7.7 ± 0.7	7.4 ± 0.4
Effluent COD (mgO_2_ L^−1^)	28.7 ± 6.7	34.2 ± 6.8	24.6 ± 3.6	25.0 ± 8.7
Effluent TSS (mg L^−1^)	5.1 ± 2.7	9.4 ± 6.1	4.9 ± 1.6	7.0 ± 7.9
Effluent turbidity (NTU)	1.7 ± 1.0	4.1 ± 3.5	1.2 ± 0.8	1.8 ± 2.9
Cleaning requirements (d^−1^) (*)	0.39	0.42	0.20	0.20

(*) Overall cleaning executed divided by the duration of corresponding period.

**Table 4:** *p*-values of the Mann–Whitney U test executed to steady state permeate turbidity data of different runs (*p*-value < 0.05 can be considered significant). For each pair of runs, four pairs of datasets were compared, i.e., the entire group of data (“all data”) and three classes distinguished according to the time between mesh cleaning and sampling.

	Compared Runs (Variables Changed)	Compared Dataset			
		All Data	1st Day	2nd Day	>2 Days
1	R20_15 vs. R50_15 (pore size)	<0.01	<0.01	0.293	<0.01
2	R50_50 vs. R100_50 (pore size)	0.016	0.013	0.285	0.327
3	R50_15 vs. R100_50 (pore size and SRT)	<0.01	0.395	0.025	<0.01
4	R20_15 vs. R50_50 (pore size and SRT)	<0.01	0.129	0.430	0.490
5	R20_15 vs. R100_50 (pore size and SRT)	0.046	0.180	0.897	0.184

1st day: sample collection started immediately after mesh cleaning. 2nd day: sample collection started 24 h after the last mesh cleaning. >2 days: sample collection started more than 2 days after the last mesh cleaning.

**Table 5:** Overview of studies that focused on the influence of the filtering support pore size.

Pore Size (µm)	Sludge	Test Type	Influence on Productivity (Pressure and/or Flux Measures)	Influence on Effluent Quality (Turbidity or TSS)	Reference
10, 40	Anaerobic	Short filtration test (1–2 h)	3 different materials were tested. Significant differences only in one case	Not studied	[14]
10, 52, 85, 200	Anaerobic	Short filtration test (5 h)	No significant differences (*p*-value > 0.05)	No significant differences (*p*-value > 0.05)	[11]
10, 52, 85, 200	Anoxic-aerobic	Long-term operation (120 d)	Lower productivity at smaller pore size	Lower quality at larger pore size	[12]
1, 5, 10, 25, 50	Aerobic	Short filtration test (70 h)	Significantly lower productivity only with 1 and 5 µm	Significantly lower quality for 25 and 50 µm	[13]
5, 10, 25	Aerobic	Long-term operation (120 d)	Lower productivity at smaller pore size	No significant differences	[13]
20, 40, 60	Aerobic	Short-term operation (4 d)	No significant differences	Lower quality at bigger pore size	[29]
20, 50	Aerobic	Long-term operation (60 d)	Lower productivity at smaller pore size	3 different air scouring rates were tested. Significant differences (*p*-value < 0.05) in 2 cases	[10]
20, 50, 100	Aerobic	Long-term operation (141–293 d)	No significant differences	Lower quality at larger pore size	This study

## Data Availability

No new data were created or analyzed in this study. Data sharing is not applicable to this article.

## Supplementary Materials

The following are available online at https://www.mdpi.com/1660-4601/18/4/1472/s1, Figure S1: Flat sheet configuration of the nylon support in the SFD MBR module; Figure S2: Mature dynamic membrane; Figure S3: Samples of (a) feed and (b) permeate; Figure S4: Permeate tank.
