# Supplementary material for: Role of Mesh Pore Size in Dynamic Membrane Bioreactors
Source: Int J Environ Res Public Health. 2021 Feb 4;18(4):1472. doi: 10.3390/ijerph18041472 (PMC7915341; doi:10.3390/ijerph18041472)
Supplement: Supplementary file 1 [file ijerph-18-01472-s001.pdf]

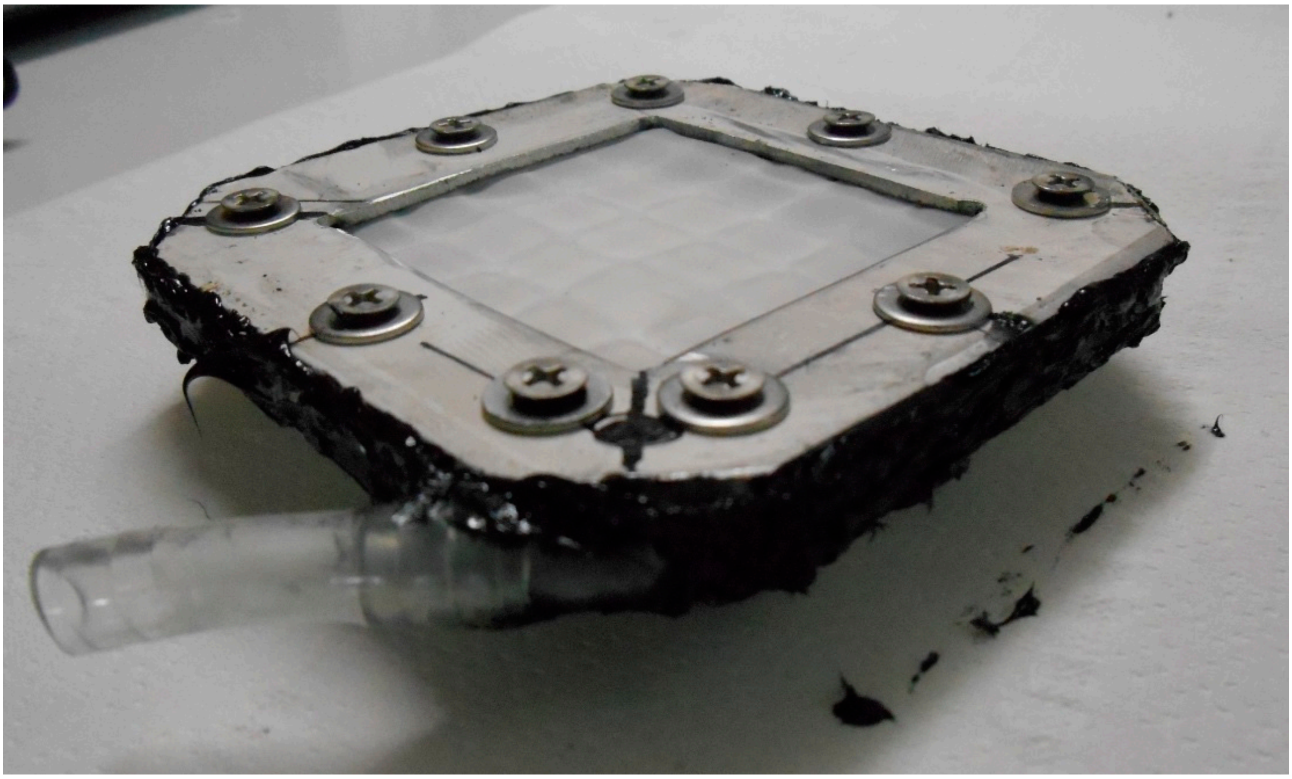

Figure S1. Flat sheet configuration of the nylon support in the SFD MBR module.

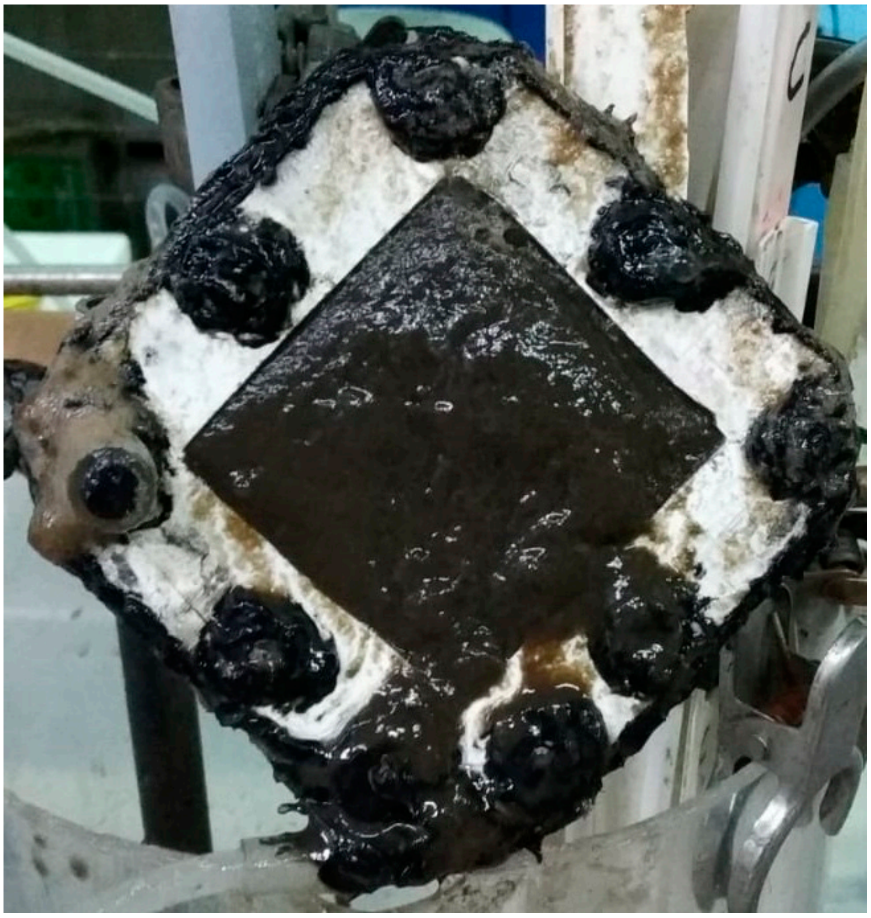

Figure S2. Mature dynamic membrane.

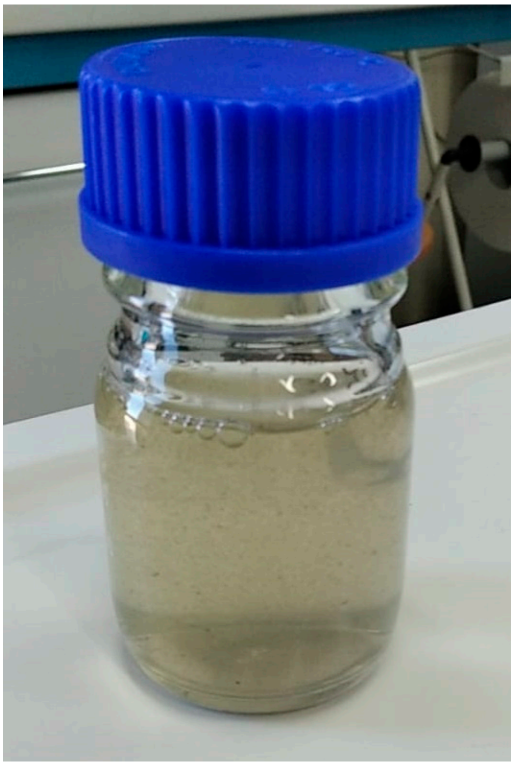

a)

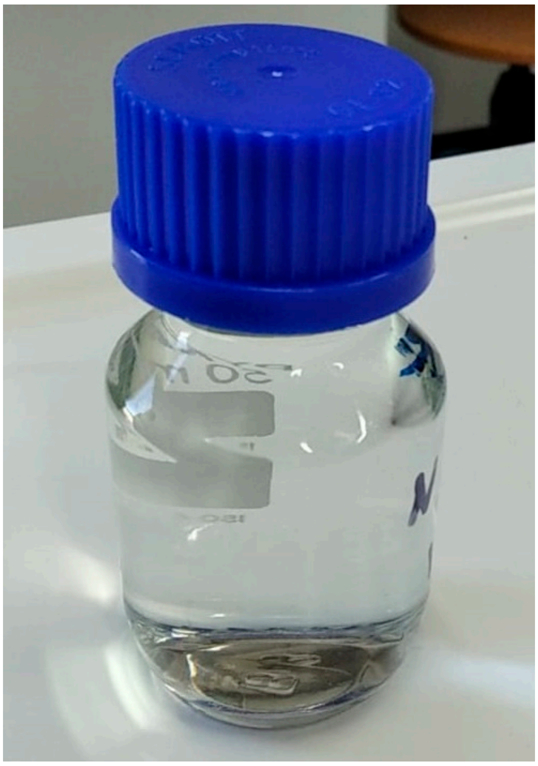

b)

Figure S3. Samples of a) feed and b) permeate.

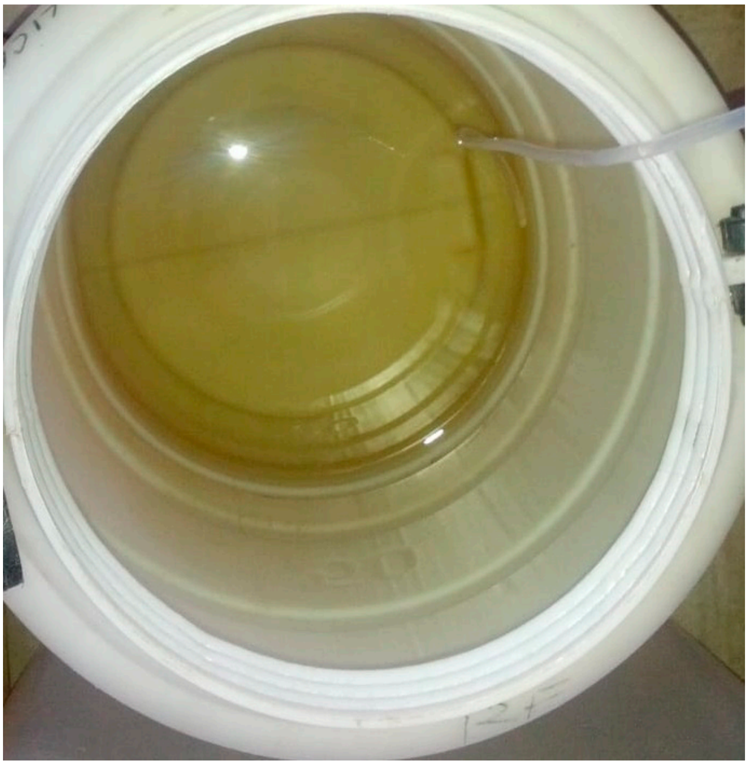

Figure S4. Permeate tank
